# New Insights into Plant Extracellular DNA. A Study in Soybean Root Extracellular Trap

**DOI:** 10.3390/cells10010069

**Published:** 2021-01-05

**Authors:** Marie Chambard, Carole Plasson, Céline Derambure, Sophie Coutant, Isabelle Tournier, Benjamin Lefranc, Jérôme Leprince, Marie-Christine Kiefer-Meyer, Azeddine Driouich, Marie-Laure Follet-Gueye, Isabelle Boulogne

**Affiliations:** 1 Normandie University, UNIROUEN, UFR des Sciences et Techniques, Glyco-MEV EA4358, SFR NORVEGE FED 4277, 76821 Mont-Saint-Aignan, France; carole.plasson@univ-rouen.fr (C.P.); marie-christine.kiefer-meyer@univ-rouen.fr (M.-C.K.-M.); azeddine.driouich@univ-rouen.fr (A.D.); marie-laure.follet-gueye@univ-rouen.fr (M.-L.F.-G.); isabelle.boulogne@univ-rouen.fr (I.B.); 2 Fédération de Recherche Normandie-Végétal, FED 4277, 76821 Mont-Saint-Aignan, France; 3 Normandy Center for Genomic and Personalized Medicine, 76000 Rouen, France; celine.derambure1@univ-rouen.fr (C.D.); sophie.coutant@inserm.fr (S.C.); isabelle.tournier@univ-rouen.fr (I.T.); 4 Plateforme de Recherche en Imagerie Cellulaire de Normandie (PRIMACEN), Normandie Université UNIROUEN, INSERM U1239, 76000 Rouen, France; benjamin.lefranc@univ-rouen.fr (B.L.); jerome.leprince@univ-rouen.fr (J.L.)

**Keywords:** plant exDNA, root extracellular trap (RET), high-throughput DNA sequencing, PEP-13, *Glycine max* (L.) Merr

## Abstract

exDNA is found in various organisms, including plants. However, plant exDNA has thus far received little attention related to its origin and role in the RET (root extracellular trap). In this study, we performed the first high-throughput genomic sequencing of plant exDNA from a *Fabaceae* with worldwide interest: soybean (*Glycine max* (L.) Merr.). The origin of this exDNA was first investigated in control condition, and the results show high-coverage on organelles (mitochondria/plastid) DNA relative to nuclear DNA, as well as a mix of coding and non-coding sequences. In the second part of this study, we investigated if exDNA release was modified during an elicitation with PEP-13 (a peptide elicitor from oomycete genus *Phytophthora*). Our results show that treatment of roots with PEP-13 does not affect the composition of exDNA.

## 1. Introduction

Extracellular DNA (exDNA) is known to be a part of different extracellular traps (ETs) in mammals and other animals like crocodiles [1], fish [2], or chickens [3]. ETs are released by immune cells like neutrophils, monocytes, mastocytes, macrophages, or eosinophils [4,5,6,7,8,9]. exDNA from ETs are released upon pathogen detection [10] or during inflammation, allergy, coagulation, or autoimmune diseases [11,12,13]. In neutrophils ET (NET), exDNA release has been well described and involves two processes: suicide lytic NETosis or vital NETosis produced by living cells [14]. In vital NETosis, exDNA may be released by mitochondria [15].

exDNA is also known to be a part of biofilms from bacteria, archaea, or fungi, allowing biofilms’ structuration and stability, and improving their resistance to antibiotics or antifungal compounds [16,17,18,19]. In soils or aquatic environments, exDNA has also been found [19,20] and used as a nutritional source for microorganisms as marine bacteria or *Archaea* [21,22].

exDNA is also a part of plant root ET (RET), a defense network located at the root apex made up of mucilaginous secretions (including glycomolecules, proteins, and specialized metabolites) and root-associated cap-derived cells, or AC-DC [23,24]. Indeed, exDNA has been highlighted in the RET of two *Fabaceae*: pea [25] and soybean [26]. This DNA might play a stuctural role in the root trap and enables to lyse bacterial cells [27]. Like NET exDNA, a study shows that plant exDNA within the RET may be synthesized and exported by living cells [28].

Although exDNA seems to be a key player in root defense [24,29], there are still many issues about its origin and modulation in the RET during plant immunity processes. A first sequence analysis was made with pea exDNA random clones in 2009 [25], but no high-throughput genomic sequencing has ever been done and, till now, no analysis has been conducted on soybean.

Herein, in order to explore the hypothesis of exDNA occurring from living cells or cell lysis as for NET [24], we provide the first DNA-seq of plant exDNA. Moreover, we investigated here if soybean root elicitation by PEP-13, a peptide from oomycete genus *Phytophthora* [30] known to induce soybean root rot, does alter RET exDNA sequence.

## 2. Materials and Methods

### 2.1. Plant Material, PEP-13 Elicitation, and exDNA Separation

*Glycine max* (L.) Merr. seeds from variety Castetis (La Dauphinoise, Vienne, France) were sterilized overnight twice in chlorine gas and grown on liquid ½ MS medium, supplemented with 2 µg/mL of peptide PEP-13 for elicited plants, at 26 °C (80–90% relative humidity (RH), 16 h to 8 h day and night cycle), in a growth chamber for five days. The peptide PEP-13 (VWNQPVRGFKVYE) used for this experiment was synthesized on the facility PRIMACEN (Université de Rouen Normandie, France).

Soybean RETs were collected aseptically by manual agitation of the roots in 400 µL of sterile DNAse-free water (Water, Sterile Nuclease-free, TFS Fisher BioReagents). exDNA was separated from the root AC-DC (root-associated cap-derived cells; [24]) by centrifugation (10 min at 3000 g). Supernatant was collected and incubated for 10 min at 100 °C and then frozen. The absence of microbial contamination was surveyed on LB medium incubated at 24 and 37 °C for 48 h. After 48 h, only sterile samples were used and treated for 30 min at 60 °C with Proteinase k (Qiagen).

### 2.2. DNA Purification and Sequencing

Each sample (300 plants/sample) was concentrated to a minimal volume (~500 µL) with a speedvac (Thermo Scientific, Waltham, MA, USA). Purification with DNeasy^®^ PowerClean^®^ Pro Cleanup kit (Qiagen, Hilden, Germany) was done on 100 µL of each sample. DNA samples were sonicated for 300 s (S220-Series Covaris) to obtain 200 pb fragments. Samples were visualized with a TapeStation 2200 (Agilent, Santa Clara, CA, USA) and High Sensitivity D1000 ScreenTape System kit (Agilent). Libraries were constructed using the NEBNext^®^ Ultra™ II kit (NEB) and 1 ng of DNA and sequenced on a MISeq System (Illumina) using 2 × 150 bases paired-end sequencing (V2 Micro 300 cycles kit, Illumina) on the Service Commun de Génomique IRIB-Inserm U1245 (Rouen, France).

### 2.3. Bioinformatic Analyses

Sequenced reads were aligned to the *Glycine max* genome Wiliams 82 Assembly 4 Annotation 1 (Wm82.a4.v1) (https://phytozome.jgi.doe.gov/) using BWA Aligner (V0.7.17) and GATK (V4.0.6.0) [31] to obtain binary alignment files and indexes. These files were visualized with Integrative Genomic Viewer (V2.6.3) [32]. The coverage for total, organelles, and nuclear DNA was calculated with the following formula: X = (*n* × l)/L; where *n* = number of reads, l = reads length (Mb), and L = genome length (Mb).

Genomic sequence was divided in 1000 pb sequences and the number of reads per 1000 pb sequence was calculated with Bedtools (V2.22) [33] using the coverage command. A differential analysis was done with DESeq2 (V2.11.40.6) [34] on these 1000 pb parts. Volcano plots and read alignment analysis were made with Excel. Sequences identification was done with NCBI (National Center for Biotechnology Information) Nucleotide databank and nucleotide BLAST tool, against the “plants” organisms’ database of NCBI.

## 3. Results

### 3.1. Differences between Nuclear and Organelle DNA Coverage

**Figure 1:**
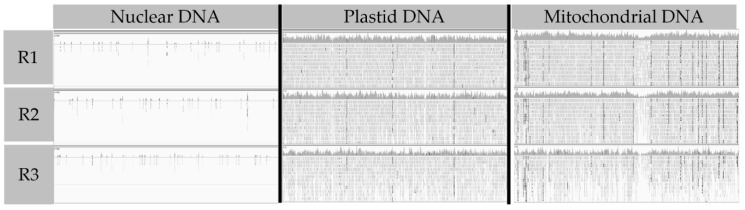
Examples of integrative genomic viewer (IGV) views of mitochondrial, plastidial, and nuclear DNA coverages with exDNA from soybean var. Castetis. For each organelle and chromosome, a representative 50 Kb part of the total sequence is shown. For nuclear DNA, the view corresponds to the position 4,527,274 pb to 4,577,274 pb on the first chromosome (representative example of all chromosomes). For plastid DNA, the view corresponds to the position 55 pb to 50,055 pb. For the mitochondrial DNA, the view corresponds to the position 36,375 pb to 86,375 pb. R = replicate.

In order to find out the origin of RET soybean exDNA, we sequenced this DNA using a next-generation sequencing approach (MiSeq, Illumina). In total, we generated 1.7 Gb reads from six samples of 300 plant RET; a total of 6.786 million reads were mapped to the soybean whole genome including nuclear (20 chromosomes) and organelles (plastid and mitochondria) sequences. For each sample, we had an average of 1,224,885 reads mapped on the whole genome. Among them, an average of 47,543 reads were mapped on organelles (mitochondria and plastid) DNA and an average of 1,177,342 reads were mapped on chromosomes. Using IGV (integrative genomic viewer), we examined the alignment of the reads (Figure 1).

Mitochondrial and plastid sequences seemed to be well and uniformly covered by exDNA reads. Furthermore, replicates did not show any differences in alignment. By contrast, nuclear sequence showed very few alignments of reads, which seemed to be randomly aligned between the different replicates.

Thus, this analysis showed a high difference in coverage of exDNA between nuclear DNA and organelles DNA. Indeed, the number of reads per mega base is surprisingly higher for plastidial and mitochondrial DNA compared with nuclear DNA (nDNA). To validate this observation, we calculated the coverage of nDNA and organelles DNA by the exDNA (Table 1).

This calculation supported our previous observations (Figure 1), indicating that the coverage is different between organelles and nuclear DNA. Actually, organelles’ coverage is more than seventy times higher for mitochondria (17.88 X) and sixty-two times higher for plastids (15.70 X) than nDNA (0.25 X).

On the other hand, it has been shown that exDNA is implicated in plant root defense [24,25,35], suggesting that it could be modified in response to pathogens or elicitors. Thus, we investigated how exDNA respond to an elicitation with PEP-13.

### 3.2. Impact of PEP-13 Elicitation on exDNA Sequence

**Figure 2:**
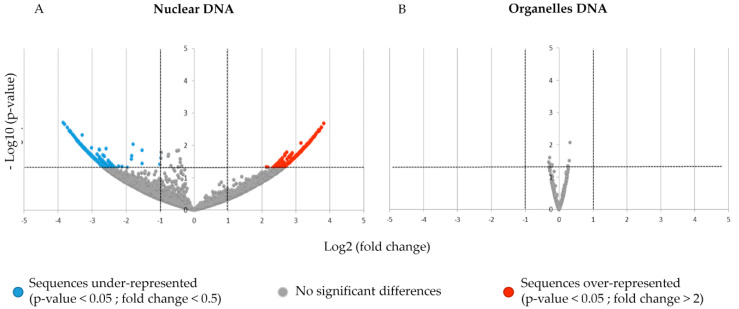
Volcano plots of exDNA from soybean var. Castetis differentially aligned in 1000 pb parts. (**A**): Nuclear DNA, (**B)**: mitochondrial and plastidial DNA merged as “organelles DNA” owing to their similarity. (**A**,**B**): 1000 pb parts of the soybean genomic sequence are represented by a point. Each point is placed according to its Log2 of fold change and the absolute value of the Log10 of its *p*-value. A color is assigned to every point depending on its fold change and *p*-value. Grey points correspond to sequences with no significant differences between PEP-13 elicited and control conditions, blue points correspond to sequences that are under-represented in control condition (fold change < 0.5 (log2 fold change < −1), *p*-value < 0.05), and red points correspond to sequences that are over-represented in PEP-13.

In order to investigate the potential changes of exDNA during a defense response, we also sequenced exDNA from elicited soybean seedlings with PEP-13, a peptide elicitor from the oomycete *Phytophthora megasperma sp. glycinea* [36], now known as *P. sojae*. Soybean whole genome (nuclear and organelles) was divided into 1000 pb parts and exDNA sequences were aligned on these parts. Then, a differential analysis was done with DESeq2 to obtain a fold change and a *p*-value for each 1000 pb part. These results are shown in two volcano plots, one for nDNA (Figure 2A) and the other for organelles DNA (Figure 2B).

This analysis shows no significant differences in the alignment of exDNA reads on organelles DNA. By contrast, alignment on nDNA revealed some significant differences between exDNA of elicited and control seedlings (Figure 2A). Indeed, we obtained 1723 parts of 1000 pb nDNA that are over-represented (FC > 2; *p*-value < 0.05), and 2393 parts of 1000 pb nDNA that are under-represented (FC < 0.5; *p*-value < 0.05). Despite the low coverage of nDNA, it appeared that this part of exDNA could be affected by the elicitation with PEP-13. In order to better understand this potential response to PEP-13, we further investigated these differentially represented sequences (Table 2).

Here, we show twenty 1000 pb parts with the ten most important FC (over-represented, FC > 2, *p*-value < 0.05) and the ten lowest FC (under-represented, FC < 0.5, *p*-value < 0.05) (Table 2). Among these twenty 1000 pb parts, a mix of coding and non-coding sequences was found.

However, these sequences are only counted 7 to 9 times in their highest-detected condition, and 0 times in their lowest-detected condition. These counts seemed to be really low, and despite their associated *p*-value and fold change, we assume that PEP-13 elicitation has a little or no effect in terms of the number of exDNA reads alignment.

**Figure 3:**
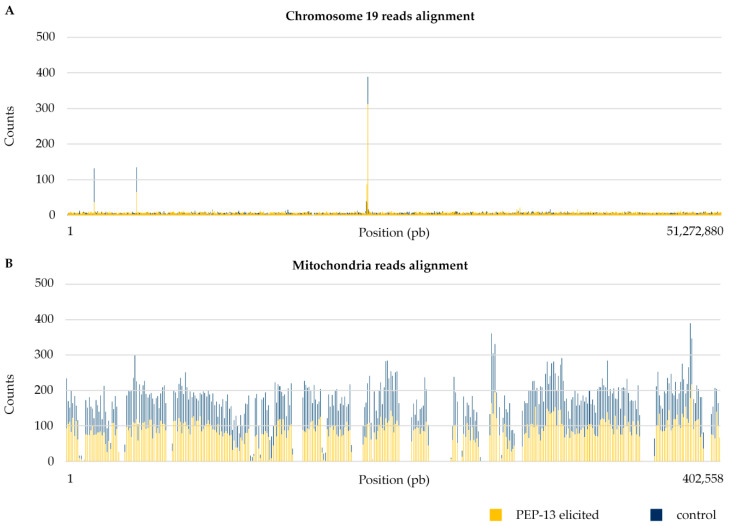
Reads’ alignment along chromosome 19 (**A**) and mitochondria (**B**) in PEP-13 elicited and control conditions. For each 1000 pb parts of the chromosome 19 and mitochondria (representative of chromosomes and organelles, respectively), the number of reads is represented on this graphic. PEP-13 elicited (**yellow**) and control condition (**dark blue**) have been overlapped in order to show their similarity. Reads were obtained from soybean var. Castetis exDNA.

In order to examine alignment profiles, we realized read alignment graphics for each chromosome and organelle. Here, we show the example of chromosome 19 (Figure 3A) and mitochondria alignment (Figure 3B), which are representative of the other chromosomes and organelles, in control (dark blue) and PEP-13 elicited (yellow) conditions. For the nuclear sequence, we highlighted a baseline of nearly zero counts all along the chromosome sequence, which is similar between the two conditions. Secondly, we can see some 1000 pb parts showing higher alignment counts (almost 400 for the 24,013,001–24,014,000 pb part in control condition), which seemed slightly lower in the elicited condition than in the control condition. This 1000 pb part was not detected previously as a differentially represented part (FC > 2; *p*-value < 0.05) because there was no significant change between PEP-13 elicited and control conditions. For organelles’ alignments, we highlighted a high baseline (around 200 counts) all along the sequence, which also seemed slightly lower in the elicited condition compared with the control condition. According to the previous differential analysis (Figure 2), there was no significant change between the two conditions.

A total of eleven 1000 pb parts from nuclear DNA with a count higher than 100 in every samples (three PEP-13 elicited and three control conditions) were found in the soybean genome. These parts with a higher alignment of reads are detailed in Table 3.

In Table 3, we show that the eleven 1000 pb parts of nuclear DNA with a high number of reads aligned are a mix of coding and non-coding sequences. Furthermore, these sequences are mainly repeated sequences and/or identified only in soybean or *Fabaceae*.

## 4. Discussion

This study is a pioneer high-sequencing approach of plant exDNA performed in soybean RET. Sequence analysis was successively done in control condition and after PEP-13 elicitation. For the control condition, reads aligned on soybean genomic DNA (nuclear and organelles DNA) suggested that soybean RET exDNA is constitutively a mix of coding and non-coding sequences from nDNA and organelles DNA. A very similar result was observed [25] with some identified sequences of pea exDNA.

In our study, we registered a higher number of reads aligned on nDNA than on mitochondrial/plastidial DNA. Nevertheless, considering the size of each genomic DNA (942.21 Mb for nuclear DNA and 0.55 Mb for organelles DNA), we show in control condition that the coverage is seventy times higher for mitochondria and plastids (17.29 X) compared with nDNA (0.25 X). This difference might be explained by the number of organelles in plant cells, and especially in root AC-DC, which appears to contains a high number of organelles [40,41]. A sequencing of roots’ AC-DC cellular DNA (including both nuclear and organelles DNA) might indicate if the number of organelles in these cells could itself explain this coverage difference.

Moreover, two other assumptions could also explain this high difference; that is, organelles DNA could be better preserved in the RET than nDNA or the major origin of RET extracellular DNA could be from organelles.

In favor of the first hypothesis, it is known that DNA degradation in the soil is dependent on extrinsic and intrinsic properties [42]. In our experimental design, as nuclear and organelles DNA sequences are in the same extracellular matrix (the RET), we assumed that extrinsic properties are not responsible for the preservation differences of exDNA. Among intrinsic properties that could affect DNA persistence in the RET, percentage of GC, DNA molecular weight and conformation, or methylation could occur [42,43,44].

When comparing the GC% of nDNA (35% GC; [45]), mitochondrial DNA (45% GC; [46]), and plastidial DNA (34% GC; [47]) sequences, some differences appear between nDNA and mitochondrial DNA GC%, but not between nDNA and plastidial DNA. Thus, the GC% intrinsic property could explain a better persistence of mitochondrial DNA, but not of plastidial DNA, in comparison with nDNA.

DNA molecular weight, including sequence length, may allow a better stability of exDNA. Molecular weight is directly correlated to soil adsorption of DNA [48]. In the RET and in our in vitro conditions, exDNA could be adsorbed by a positively charged surface [49] or a positively charged component of the RET like histones [50], increasing its conservation. Currently, there is no study comparing exDNA sequence sizes depending on their origin (nuclear or organelles DNA), which could explain the differences of nDNA and organelles DNA conservation.

Another explanation of these conservation differences could be the fact that plastids and mitochondrial DNA are both circular DNA, in contrast with nDNA, which is linear. Indeed, circular DNA is known to be more stable than linear DNA [43]. Regarding this property, organelles DNA degradation in the RET might be longer than nDNA degradation, resulting in these conservation differences.

ExDNA could be fragmented during cellular lysis of root tip cells as a result of DNAse activity. It is known that DNAse activity could be both enhanced or inhibited by DNA methylation in a misunderstood way [51]. Thus, a difference in DNA methylation between chromosomes and organelles could explain conservation differences. In plants, DNA methylation is species- and organelles-specific [52,53]. Two major methylation types are found in plants DNA: m5C (5-methylcytosine) and m6A (N6-methyladenine). In mitochondrial DNA, only m6A is found, unlike plastids and nuclear DNA, which contain both of these types of methylation. Moreover, recently, m6A methylation density has been investigated in soybean, showing 1.4% for plastid DNA, 1.05% for mitochondrial DNA, and only 0.05% for nDNA [54]. Given this hypothesis, an exDNA methylation analysis might be done in order to understand if methylation processes are involved in the conservation of exDNA sequences.

Our second hypothesis is that exDNA could mainly originate from mitochondrial and plastids. Indeed, it has been shown in NET that exDNA could originate from mitochondria [15,55]. In this case, NET formation does not induce neutrophil death (vital NETosis) [14]. It is thus possible to imagine that RET formation could consist of the programmed DNA release from organelles. In this assumption, mitochondrial and plastidial DNA would be released in the extracellular matrix without being degraded during cell death. This could explain the high conservation and coverage of organelles DNA. Nuclear DNA in the RET, for its part, could be explained by the release of fragmented DNA during the death of the older root cells. Furthermore, this hypothesis is consistent with the results obtained in the literature [25]. Indeed, they observed that exDNA release in pea RET seemed to be newly synthetized DNA. Moreover, plastids division is not dependent on the cell cycle [56] and could occur regularly (from 1 to 4 h in wheat) [57] thanks to specific regulation mechanisms [58,59].

Considering nDNA, we recorded a particular profile of read alignment (Table 3). While read alignment showed a low coverage on chromosomes, eleven 1000 pb parts had a significantly higher number of counts (>100 counts for each replicate and experimental condition). Thus, it seemed that some parts of the nDNA are better conserved than others. Among these 1000 pb parts, we found sequences that are specific to the soybean or Fabaceae genomes. Among these, three are also involved in plant defense response, which strengthened the importance of exDNA in root defense. We also found several repeated sequences with a higher read depth. This could reflect a variation in the copy number of the soybean variety used in this study compared with the reference genome used [60]. Indeed, the soybean variety used for this study (Castetis) was not the same as the genome reference variety (Williams 82). Thus, it could be possible that Castetis has more repeated sequences in its genome as a result of varietal selection. Indeed, pathogen or lodging resistances are significant criteria for varietal selection because of the sub-functionalization reducing the gene pleiotropy and responsible for duplication of genes [61,62].

Another explanation of the high number of these reads is that these sequences could be recognized as DAMPs (damage-associated molecular patterns). Indeed, plant genomic DNA is a DAMP and plants seem to be able to recognize its own DNA among the DNA of another species [63]. In our study, we identified some sequences specific of the *Glycine max* genome (Table 3). These sequences could be recognized by the root as DAMPs in the RET and initiate a plant defense response, improving plant resistance to pathogens, making exDNA an endogenous elicitor. In order to confirm this hypothesis, it might be interesting to test if these exDNA sequences are able to induce plant defense response.

The second part of our study was to observe exDNA during a defense response to PEP-13 elicitation. Even if our differential analysis seemed to show some differences for the nDNA sequences, we assume that it was not a significant variation, considering the really low counts of these sequences. Two hypotheses could explain these results: exDNA alignment might not be influenced by PEP-13 elicitation, or exDNA might not be affected by any elicitation. Regarding these results and the hypothesis, elicitation tests should be done with other elicitors (β-glucan elicitor, others peptides, lipopolysaccharides, and so on) in order to find out if exDNA alignment could be influenced by elicitation.

In summary, our findings reveal that exDNA is constitutively a mix of coding and non-coding sequences from mainly organelles DNA and, in the minority, from nuclear DNA, which are not modified by PEP-13 elicitation. Further investigations are now needed to know if methylation processes are involved in the conservation of exDNA sequences, if specific nuclear sequences of exDNA could be recognized by the root as a DAMP, or if other MAMPs could significantly alter RET exDNA sequences.

## Figures and Tables

**Table 1:** Soybean variety Castetis extracellular DNA counts and coverage for nuclear DNA, mitochondrial DNA, plastidial DNA, and total DNA. Coverage has been calculated according to the formula explained in Material and Methods section.

Cellular Structure	exDNA	
	Counts Mean	Coverage (X)
Nuclear DNA	1,177,342.00	0.25
Mitochondrial DNA	35,765.33	17.88
Plastidial DNA	11,777.67	15.70

**Table 2:** Identification of differentially represented 1000 pb parts of nuclear DNA in exDNA from soybean var. Castetis. E = PEP-13 elicited condition; NE = control condition; N/A = non-coding sequence.

Chromosome	Position (pb)		Condition	Counts Mean	Log2 (FC)	*p*-Value	Identification in Soybean or Blast (% Identity)
	START	STOP					
Gm07	26,655,001	27,656,000	E	8	3.8293	0.0021	PREDICTED: *Glycine max* putative dual specificity protein phosphatase DSP8
			NE	0			
Gm15	15,541,001	15,542,000	E	8	3.7595	0.0028	N/A
			NE	0			
Gm07	7,109,001	7,110,000	E	7	3.7505	0.0028	N/A
			NE	0			
Gm09	27,858,001	27,859,000	E	7	3.7060	0.0033	85.88% *Glycine max* retrotransposon gmw1-45m6-re-2
			NE	0			
Gm16	14,656,001	14,657,000	E	7	3.6978	0.0034	87.13% PREDICTED: *Glycine soja* organic cation/carnitine transporter 7-like
			NE	0			
Gm05	2,829,001	2,830,000	E	7	3.6796	0.0035	N/A
			NE	0			
Gm08	12,008,001	12,009,000	E	7	3.6777	0.0036	Auxin-induced protein 6B-like
			NE	0			
Gm01	51,034,001	51,035,000	E	7	3.6873	0.0036	Phosphopantethine adenyltransferase
			NE	0			
Gm13	23,974,001	23,975,000	E	7	3.6657	0.0037	60S ribosomal protein L23
			NE	0			
Gm14	46,813,001	46,814,000	E	7	3.6904	0.0020	N/A
			NE	0			
Gm20	45,220,001	45,221,000	E	0	−3.8522	0.0022	N/A
			NE	9			
Gm07	22,405,001	22,406,000	E	0	−3.8070	0.0022	N/A
			NE	8			
Gm13	15,882,001	15,883,000	E	0	−3.7247	0.0029	N/A
			NE	8			
Gm14	20,131,001	20,132,000	E	0	−3.6824	0.0036	N/A
			NE	8			
Gm01	29,642,001	29,643,000	E	0	−3.6575	0.0036	N/A
			NE	7			
Gm19	26,749,001	26,750,000	E	0	−3.6457	0.0038	N/A
			NE	7			
Gm09	46,814,001	46,815,000	E	0	−3.6416	0.0037	TPR (tetratricopeptide) repeat-containing protein ZIP4-like
			NE	7			
Gm15	28,346,001	28,347,000	E	0	−3.6416	0.0037	N/A
			NE	7			
Gm12	19,408,001	19,409,000	E	0	−3.6362	0.0040	N/A
			NE	7			
Gm16	127,001	128,000	E	0	−3.6279	0.0040	Shaggy-related protein kinase kappa-like
			NE	4			

**Table 3:** Identification of 1000 pb parts of nuclear DNA in exDNA from soybean var. Castetis with a higher read alignment (count > 100). N/A = non-coding sequence.

Chromosome	Position (pb)		Identification in Soybean or Blast (% Identity)	Comment
	START	STOP		
Gm01	15,120,001	15,121,000	N/A	Repeated sequence, identified only in *Glycine max*
Gm02	21,073,001	21,077,000	LOC100785390 protein kinase and PP2C-like (protein phosphatase 2C) domain-containing protein [*Glycine max* (soybean)]	Kinases involved in plant defense response [37]
GM04	26,981,001	26,983,000	N/A	Repeated sequence, identified only in *Glycine max* and *Glycine soja*
Gm09	25,428,001	25,429,000	79.66% *Pisum sativum* clone Ps-phage20 Ogre retrotransposon, partial sequence	Identified only in *Fabaceae*
Gm09	23,598,001	23,599,000	96.05% *Glycine max* NB-LRR (nucleotide binding—leucine rich repeat) type disease resistance protein Rps1-k-1 (Rps1-k-1) and NB-LRR type disease resistance protein Rps1-k-2 (Rps1-k-2)	Repeated sequence, involved in plant defense against *Phytophthora sojae*. Might be near or a part of heterochromatic DNA [38]. Identified only in *Glycine max*
Gm10	21,046,001	21,047,000	93.01% PREDICTED: *Glycine soja* probable WRKY transcription factor 11 (LOC114391634), mRNA	Transcription factor WRKY involved in defense response [39]. Identified only in *Glycine max*
Gm17	25,874,001	25,877,000	97.96% PREDICTED: *Glycine soja* L-ascorbate oxidase homolog (LOC114411184), mRNA	Identified in *Fabaceae* and *malvaceae* (*Gossypium*)
Gm18	32,363,001	32,364,000	72.09% PREDICTED: *Glycine soja* uncharacterized LOC114387464, ncRNA	Repeated sequence, identified only in *Glycine max*
Gm18	32,392,001	32,393,000	N/A	Repeated sequence
Gm19	24,013,001	24,014,000	Triacyl-glycerol lipase 2	Identified only in *Fabaceae*
Gm20	20,695,001	20,697,000	N/A	Repeated sequence, identified only in *Glycine max*

## Data Availability

The data presented in this study are openly available in the European Nucleotide Archive (ENA) at http://www.ebi.ac.uk/ena/data/view/PRJEB42337.
